# A study of deep learning methods for de-identification of clinical notes in cross-institute settings

**DOI:** 10.1186/s12911-019-0935-4

**Published:** 2019-12-05

**Authors:** Xi Yang, Tianchen Lyu, Qian Li, Chih-Yin Lee, Jiang Bian, William R. Hogan, Yonghui Wu

**Affiliations:** Department of Health Outcomes and Biomedical Informatics, College of Medicine, University of Florida, Clinical and Translational Research Building 2004 Mowry Road, PO Box 100177, Gainesville, Florida USA

**Keywords:** EHR, Protected health information, De-identification, Cross institutions, Deep learning

## Abstract

**Background:**

De-identification is a critical technology to facilitate the use of unstructured clinical text while protecting patient privacy and confidentiality. The clinical natural language processing (NLP) community has invested great efforts in developing methods and corpora for de-identification of clinical notes. These annotated corpora are valuable resources for developing automated systems to de-identify clinical text at local hospitals. However, existing studies often utilized training and test data collected from the same institution. There are few studies to explore automated de-identification under cross-institute settings. The goal of this study is to examine deep learning-based de-identification methods at a cross-institute setting, identify the bottlenecks, and provide potential solutions.

**Methods:**

We created a de-identification corpus using a total 500 clinical notes from the University of Florida (UF) Health, developed deep learning-based de-identification models using 2014 i2b2/UTHealth corpus, and evaluated the performance using UF corpus. We compared five different word embeddings trained from the general English text, clinical text, and biomedical literature, explored lexical and linguistic features, and compared two strategies to customize the deep learning models using UF notes and resources.

**Results:**

Pre-trained word embeddings using a general English corpus achieved better performance than embeddings from de-identified clinical text and biomedical literature. The performance of deep learning models trained using only i2b2 corpus significantly dropped (strict and relax F1 scores dropped from 0.9547 and 0.9646 to 0.8568 and 0.8958) when applied to another corpus annotated at UF Health. Linguistic features could further improve the performance of de-identification in cross-institute settings. After customizing the models using UF notes and resource, the best model achieved the strict and relaxed F1 scores of 0.9288 and 0.9584, respectively.

**Conclusions:**

It is necessary to customize de-identification models using local clinical text and other resources when applied in cross-institute settings. Fine-tuning is a potential solution to re-use pre-trained parameters and reduce the training time to customize deep learning-based de-identification models trained using clinical corpus from a different institution.

## Background

Unstructured clinical text has been increasingly used in clinical and translational research as it contains detailed patient information that not readily available in structured medical codes. De-identification [1] is a critical technology to facilitate the use of clinical narratives while protecting patient privacy and confidentiality [2]. The Health Insurance Portability and Accountability Act (HIPAA) “Safe Harbor” rules identified 18 Protected Health Information (PHI) to be removed to generate de-identified copy of clinical data [3]. As manually de-identification is often time consuming and not applicable to large volumes of clinical text, researchers have developed natural language processing (NLP) methods to automatically identify and remove PHIs from clinical notes [4, 5]. The clinical NLP community has invested great efforts in developing statistical NLP methods for de-identification of clinical notes. Many state-of-the-art NLP methods for de-identification are based on supervised machine-learning methods [1, 6]. Several de-identification corpora have been annotated to support the training of supervised machine learning methods [7–10]. These annotated corpora are valuable resource to develop automated clinical NLP systems for de-identification of clinical text at local hospitals. However, most existing studies on de-identification of clinical text were conducted in a single-institute setting, where the training data and test data were from the same institution. Up until now, there is limited study to explore automated de-identification of clinical notes under cross-institute settings [11–13].

Most studies approach the de-identification as a clinical named entity recognition (NER) [14] task, which is a standard clinical NLP task to identify medical concepts and determine their semantic categories. The two tasks are very similar to each other as both focus on the identification of information of interests and clinical NER methods can be applied for de-identification. However, there are several differences between the two tasks. First, the de-identification task usually includes more semantic categories than traditional clinical NLP. Second, the de-identification task usually focuses on patient privacy information such as patients’ names, phone numbers, and ID numbers, whereas, traditional clinical NLP tasks often focus on medical concepts such as problems, diagnoses and medications. Third, identify the information is much important than determine the semantic category in de-identification of clinical notes as the goal of de-identification is to remove PHIs. The clinical NLP community has organized several shared tasks to assess the current clinical NLP systems on de-identification of clinical text. The i2b2 (Informatics for Integrating Biology and the Bedside) organized clinical NLP challenges [7, 15] in 2006 and 2014 with de-identification tracks focused on identifying PHI from clinical narratives. The i2b2 2006 challenge developed a corpus consists of 889 de-identified records, collected in one record per patient manner and the i2b2 2014 challenge further extended the challenge using 1304 clinical notes from 296 diabetic patients. The organizers manually identified the PHIs and replace them with realistic surrogates for challenges. In 2016, the Centers of Excellence in Genomic Science (CEGS) and Neuropsychiatric Genome-Scale and RDOC Individualized Domains (N-GRID) also organized a shared task on de-identification of a new corpus of 1000 psychiatric notes [10]. The results released through the challenges show that the participated NLP systems performed quite well on identify PHIs from clinical narratives. For example, the best performance achieved in the 2014 i2b2 challenge is around .95 or slightly higher. In these challenges, the evaluation of de-identification is conducted using training and testing data from the same institutes.

Researchers have applied various methods for de-identification of clinical notes, including rule-based methods, machine learning-based methods, and hybrid methods that combine both approaches. In rule-based methods, researchers manually curated rules and used medical vocabularies to match common patterns of PHIs. Usually, regular expression was used to implement the rules. The rule-based methods are straightforward and easy to adjust. However, the development of rule-based systems is time consuming and may not generalizable to clinical notes with different patterns. Most state-of-the-art de-identification systems are based on supervised machine learning methods or hybrid methods. Machine learning methods approach the de-identification as a sequence labeling problem, where a computational model is developed to label the input word sequence with predefined labels (e.g., ‘BIO’ format labels). Researchers have applied many machine learning-based clinical NER methods including Conditional Random Fields (CRFs) [16], Maximum Entropy (ME), and Structured Support Vector Machines (SSVMs) [17] for de-identification. Machine learning-based de-identification methods requires a training set with all PHIs manually labeled. To develop machine learning models, researchers extracted different linguistic features (e.g., morphology of words, syntactic information such as part-of-speech) and various lexical features (e.g., word case and special symbols) from the clinical text. Machine learning-based de-identification methods usually have a better generalizability to new clinical text. Therefore, they perform better to identify PHIs that not covered by existing dictionaries compared with rule-based methods. Machine learning methods achieved state-of-the-art performance in a number of NLP challenges on de-identification. For example, the best de-identification system in 2014 i2b2 challenge (team from Nottingham) developed a CRFs model and combined it with a rule-based post-processing pipeline based on regular expression and dictionaries [18]. The second-best system in this challenge also developed a CRFs model and combined it with a rule-based pipeline to identify standard PHIs such as PHONE, FAX, and MEDICAL RECORD NUMBER [19]. In our previous study, we also developed a CRFs model with a rule-based post-processing pipeline, which achieved the second-best performance in 2016 CEGS N-GRID shared task on de-identification of psychiatric notes [20]. A critical step of developing machine learning-based de-identification systems is to extract useful features. Researchers have examined various features such as linguistic feature, dictionary lookup, unsupervised clustering, and distributed word representations.

Recently, deep learning models have been applied to NER and de-identification and demonstrated better performance in the clinical domain [21, 22]. A break through in deep learning-based NLP methods is the distributed word representation trained using word embedding algorithms. Previous studies have demonstrated that word embeddings algorithms could capture various features in a low-dimension matrix, thus alleviated the researchers from time consuming feature engineering. Deep learning models based on recurrent neural networks (RNN) and convolutional neural networks (CNN) have been widely used for clinical NER and de-identification of clinical notes. We have explored CNNs and RNNs for standard clinical NER in our previous work [23–26]. Recent studies reported an RNN model implemented using the long-short term memory strategy and a CRFs layer (LSTM-CRFs) achieved superior performance for de-identification. For example, Liu et al. [27] developed a LSTM-CRFs model with a rule-based post-processing pipeline, which outperformed the best CRFs model developed during the 2014 i2b2 challenge. Dernoncourt et al. [28] also applied a similar LSTM-CRFs model for de-identification of clinical notes. Most of the previous de-identification studies in the clinical domain utilized training and test data from the same institution for training and evaluation. There are few studies to examine the state-of-the-art deep learning models in cross-institution settings [6].

In this study, we examined methods to customize a deep learning-based method, LSTM-CRFs, for de-identification of clinical notes at UF Health. We developed the de-identification models using a clinical corpus developed by the 2014 i2b2/UTHealth challenge and evaluated the performance using clinical notes collected from UF Health. Then, we customized the LSTM-CRFs model using local notes and other resources and compared the performance. We also compared five different word embeddings trained from the general English text, de-identified clinical text, and biomedical literature. To the best of our knowledge, this is one of the earliest studies to customize deep learning-based de-identification methods at cross-institution settings.

## Materials and methods

### Data sets

In this study, we used clinical notes from the 2014 i2b2/UTHealth challenge and UF Health Integrated Data Repository (IDR). The i2b2/UTHealth corpus was extracted from the Research Patient Data Repository of Partners Healthcare [15]. The released dataset contains a total number of 1304 clinical notes from 296 patients. We split the dataset into a training set of 997 notes (3/4 of the total, denoted as i2b2 training) and a validation set of 325 notes (1/4 of the total, denoted as i2b2 validation). The UF Health IDR is a secure, clinical data warehouse (CDW) that aggregates data from the university’s various clinical and administrative information systems, including the Epic electronic medical record (EMR) system. As of February 2019, the IDR contains data for encounters that occurred after June 2011, with a total of more than 1105 million observational facts pertaining to 1.17 million patients. For cross-institute evaluation, we randomly collected a total number of 4996 clinical notes from the UF Health IDR. These clinical notes were from 97 patients and distributed in 39 different note types. The top 3 most common note types include PROGRESS NOTES, RADIOLOGY REPORT, and H&P (i.e., History and Physical Examination). We randomly selected 500 notes from the UF Health dataset using stratified sampling based on the note types. Three annotators (TL, QL and CL) manually annotated the PHIs from the 500 notes. We used 200 notes as the test set (denoted as UF test) and reserved the rest as the datasets for training (a total of 233 notes, denoted as UF training) and validation (a total of 77 notes, denoted as UF validation).

The i2b2/UTHealth corpus followed annotation guidelines developed by Stubbs et al. based on an extension of the HIPPA guidelines [9]. To facilitate cross institution analysis, we adjusted the annotation guideline and merged several rare PHIs for the annotation of UF Health corpus: (1) excluded the *days of week*, *seasons* and *holidays*, *state* and *country* as they are not required by HIPPA; (2) merged the *phone* and *fax* as PHONE; (3) merged *email*, *URL* and *IP Address* as WEB; (4) merged organization and hospital as INSTITUTE. We adjusted the PHI annotations in the 2014 i2b2/UTHealth corpus to make the annotations consistent. Table 1 shows the distribution of different PHI categories in i2b2/UTHealth corpus and UF Health corpus.

**Fig. 1:**
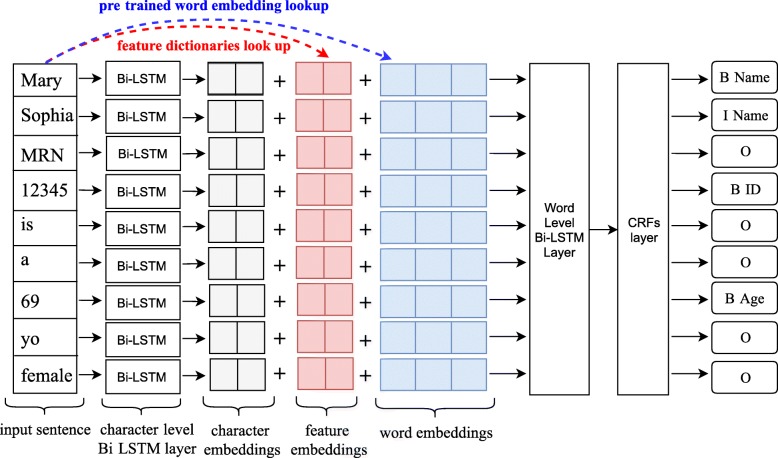
An overview of the LSTM-CRFs model with knowledge-based features derived from the local resources

**Table 1:** PHI distributions in the 2014 i2b2/UTHealth de-identification corpus and UF Health clinical notes

PHI Category	Number of Annotations				
	2014 i2b2/UTHealth		UF Heath		
	Training	Validation	Training	Validation	Test
DATE	9067	3104	2056	774	1872
NAME	5472	1868	856	356	771
AGE	1507	490	158	86	164
ID	1142	364	156	41	137
PHONE	406	128	50	28	47
WEB	6	1	0	0	4
INSTITUTE	1926	592	128	72	119
STREET	280	72	25	6	21
CITY	502	152	43	26	45
ZIP	276	76	34	11	20
Total	20,584	6847	3506	1400	3200

### Pre-processing clinical notes

We developed a pre-processing pipeline to perform typographic error correction and text normalization. The most common typographic errors are missing spaces between two words. For example, the token “prnInsulin” should be split into two words including “prn” and “Insulin”. We developed a set of heuristic rules to perform error correction. We also performed standard NLP preprocessing procedures such as sentence boundary detection and word tokenization. The BIO tagging schema [29] was used to represent PHIs.

### Deep learning model for de-identification

In this study, we adopted the LSTM-CRFs model for de-identification as it achieved superior performance compared with other ML-based methods. To incorporate features from local vocabulary, we utilized a feature embedding layer to incorporate linguistic and knowledge-based features with character and word embeddings [25]. We extracted two most important linguistic features, part-of-speech and word shape, according to previous works [27, 30, 31]. Knowledge-based features are derived from local vocabulary, which is different from the word embeddings that derived from unlabeled clinical text. Fig. 1 shows an overview of the architecture.

### Word embeddings

As a previous study [32] demonstrated that word embeddings have remarkable impact for deep learning-based NLP methods, thus, we examined five different word embeddings trained with different algorithms and corpora for de-identification. The five embeddings are: 1) GoogleNews embeddings – developed by google using the word2vec on part of the Google news dataset [33]; 2) CommonCrawl embeddings – released by Facebook trained using the fastText [34] on the Common Crawl dataset [35]; 3) MIMIC-word2vec – trained using clinical notes from the Medical Information Mart for Intensive Care III (MIMIC-III) database [36] using word2vec; 4) MIMIC-fastText – trained using clinical notes from MIMIC-III with the fastText algorithm; 5) MADE embeddings – developed by Jagannatha et al. using the skip-gram method on a combined corpus of PubMed open access articles, English Wikipedia and an unlabeled corpus of around hundred thousand Electronic Health Records [37].

### Customizing using UF clinical notes

We sought to customize i2b2 models (models trained using only i2b2 data) using UF clinical notes. We compared two different strategies to customize the i2b2 models: 1) merge UF training set with i2b2 training set and retrain the model from scratch; and 2) fine tune the i2b2 models using UF training set. The first strategy is straightforward – we simply merge the notes and retrain new models. In the second strategy, we reused the i2b2 models and continue training them using UF notes – “fine-tuning”. Fine-tuning is a key technology to enhance deep learning-based NLP model performances on various tasks [38–40]. Instead of training from scratch (where the parameters are randomly initialized), the fine tuning is based on pretrained weights from an existing model (i.e., i2b2 models). Therefore, the training time can be reduced. For comparison, we also developed a LSTM-CRFs model using only the UF training set.

### Knowledge-based feature as embeddings

We used existing dictionaries of U.S. city names and zip codes from *Encyclopedia Britannica* (https://www.britannica.com/), general first and last names from data.world (https://data.world/), and people’s names and health provider names at UF Health as a knowledge base for PHIs. To use the existing knowledge, we extract the semantic categories (e.g., CITY, NAME), matching boundaries (represented using BIO), and matching conditions (exact or partial) as features using a fuzzy matching dictionary lookup. Our previous study [25] has proved that the knowledge-based feature embedding layer improved the performance of clinical NER by integrating knowledge features with word embeddings. Chen et al. [27] and Jiang et al. [30] both showed that the knowledge-based features as complimentary resources to word embeddings improved the performance of identifying PHIs.

### Experiments and evaluation

We used a LSTM-CRFs model developed in our previous work [25] using Tensorflow [41]. We trained LSTM-CRFs models using the training set and optimized parameters and selected the best word embeddings according to performance on the validation set. The optimized LSTM-CRFs model used the following parameters: the word embedding dimension was 300; the character embedding dimension was 25; the bidirectional word-level LSTM had an output dimension of 100; and the bidirectional character-level LSTM had an output size of 25; the learning rate was fixed at 0.005; the input layer for the word-level LSTM applied a dropout at probability of 0.5; the stochastic gradient descending applied a gradient clapping at [− 5.0, 5.0] and a momentum term fixed at 0.9. In the training from scratch experiments, the number of training epochs was set to 30. For fine tuning, the number of training epochs was set to 15. We did not apply early stop strategy in any of the experiments. We compared performance of LSTM-CRFs models with or without knowledge base features. For the models with a knowledge feature embedding layer, the best embedding dimension for sematic features (i.e., city, zip code, names) was 20 and for lexical features (i.e., part-of-speech tagging, word shape) was 15, respectively. For evaluation, we reported the micro-averaged strict and relax precision, recall, and F1-score.

## Results

Three annotators annotated 8106 PHIs from 500 UF Health notes with an inter-annotator agreement of 0.889. Table 1 compares detailed number of PHIs between UF data and i2b2/UTHealth corpus. Table 2 compares the performance of LSTM-CRFs model on i2b2 validation set using different word embeddings. The model trained with the CommonCrawl embeddings achieved the best strict and relax F1 scores of 0.9547 and 0.9646, respectively, outperforming other embeddings. Therefore, we used the CommonCrawl as the word embeddings for the rest of the experiments.

**Table 2:** Performance of LSTM-CRFs trained with different word embeddings (trained using i2b2 training set and evaluated using i2b2 validation set)

Model	Embedding	Performance on validation set (i2b2/UTHealth)					
		Strict			Relax		
		Precision	Recall	F1 score	Precision	Recall	F1 score
LSTM-CRFs	GoogleNews	0.9679	0.9263	0.9466	0.9783	0.9362	0.9567
	CommonCrawl	0.9697	0.9401	**0.9547**	0.9797	0.9498	**0.9646**
	MIMIC-word2vec	0.9669	0.9341	0.9502	0.9774	0.9443	0.9606
	MIMIC-fastText	0.9631	0.9380	0.9504	0.9758	0.9504	0.9629
	MADE	0.9662	0.9158	0.9403	0.9782	0.9271	0.9520

Best F1 scores are highlighted in bold

Table 3 compares the performance of the LSTM-CRFs models trained only using the i2b2 data with the new models that customized using UF data. Compared to the LSTM-CRFs model, the models with additional lexical features and knowledge features improved the performance (i.e., F1 scores). Among the models trained only using the i2b2 data, the LSTM-CRFs model with lexical and knowledge features achieved the best strict and relax F1 scores of 0.8736 and 0.9197 on the UF test set, respectively. Using only the UF training, the best model achieved strict and relax F1 scores of 0.9195 and 0.9468, respectively, outperforming the models trained only using the i2b2 data. For the customized models, the model trained using the i2b2 data and later fine-tuned using the UF data achieved the best F1 scores of 0.9288 and 0.9584, respectively. The other customized model, trained by merging the i2b2 and UF training data, achieved a comparable performance with strict and relax F1 scores of 0.9257 and 0.9582.

**Table 3:** Performance of LSTM-CRFs models on UF test set

Model	Training data	Fine Tuning	Performance on UF Test					
			Strict			Relax		
			Pre	Rec	F1	Pre	Rec	F1
LSTM-CRFs	i2b2	NA	0.8883	0.8274	0.8568	0.9288	0.8651	0.8958
LSTM-CRFs+Lexical	i2b2	NA	0.8767	0.8509	0.8636	0.9314	0.9041	0.9175
LSTM-CRFs+Lexical + Knowledge	i2b2	NA	0.8767	0.8706	0.8736	0.9229	0.9166	0.9197
LSTM-CRFs+Lexical + Knowledge	i2b2	UF	0.9474	0.9109	**0.9288**	0.9776	0.9400	**0.9584**
LSTM-CRFs+Lexical + Knowledge	UF	NA	0.9408	0.8992	0.9195	0.9705	0.9277	0.9486
LSTM-CRFs+Lexical + Knowledge	i2b2 + UF	NA	0.9352	0.9163	0.9257	0.9681	0.9484	0.9582

Best F1 scores are highlighted in bold

Table 4 shows the performance for each PHI category achieved by the customized LSTM-CRFs model using fine-tuning. According to the results, the customized model achieved relaxed F1 scores > 0.9 for most of the PHI categories, including the best F1 score (0.9831) for DATE. On the other hand, for INSTITUTE, CITY, and STREET, the relaxed F1 scores are between 0.6 and 0.85. For the WEB, none of the four PHIs were detected.

**Table 4:** Performances for each PHI category achieved by the customized LSTM-CRFs model using fine-tuning

Entity Type	Performance on UF test set					
	Strict			Relax		
	Precision	Recall	F1 score	Precision	Recall	F1 score
DATE	0.9807	0.977	0.9789	0.985	0.9813	0.9831
AGE	0.9861	0.8659	0.9221	0.9861	0.8659	0.9221
ID	0.9173	0.8905	0.9037	0.9624	0.9343	0.9481
NAME	0.9029	0.8807	0.8917	0.9694	0.9455	0.9573
PHONE	0.9048	0.8085	0.8539	0.9762	0.8723	0.9213
ZIP	0.75	0.75	0.75	0.9	0.9	0.9
INSTITUTE	0.75	0.5042	0.603	0.9375	0.6303	0.7538
CITY	0.9048	0.4222	0.5758	1	0.4667	0.6364
STREET	0.55	0.5238	0.5366	0.85	0.8095	0.8293
WEB	0	0	0	0	0	0

### Error analysis

We performed an error analysis using the best de-identification model customized with UF data through fine-tuning and summarized them into four categories including boundary mismatch, wrong semantic category, false positives, and false negatives (missed by our system) [6]. Boundary mismatches and false negatives are more common for the NAME category. For example, our system missed the suffix “Jr.” in the NAME PHI “Xxx Yyy Jr.” (Here we de-identified the name for privacy). One possible reason for false negatives may be that the word embeddings were trained using a general English corpus, which could not cover some of the name strings. Thus, all the uncovered words were replaced as “UNKNOWN” during prediction. The wrong semantic category errors are more common for ID and PHONE PHIs. The entities in the ID category are consist of MRNs, physician IDs, Account IDs, and other unique identifiers that consist of numbers. Some Account IDs have a similar format as PHONE numbers without area codes. In addition, a few physician IDs have a similar context environment as the PHONE numbers – they often occurred after NAME PHIs. Therefore, these PHIs consist of numbers are more likely to cause wrong semantic category errors. Nevertheless, these PHIs were able to be de-identified as they were at least detected by our system, even with wrong semantic types. For false positives, we observed that the most common errors are from some lab tests with numeric results. For example, the “1/2” in “BRCA 1/2 Neg” means “BRCA type 1 and 2” but has a similar format as DATE. These false positives are not likely to expose PHIs, but they may reduce useful non-sensitive information from clinical text.

## Discussion

In this study, we examined deep learning-based de-identification methods at a cross-institute setting, where the training data and test data are from different sources. We trained models using a corpus developed by 2014 i2b2/UTHealth challenge and examined the performance using clinical notes from UF Health. We compared five pre-trained word embeddings from the general English, clinical narratives, and biomedical literature for de-identification. We also compared two strategies to customize the models using resources from UF Health. The experimental results show that the LSTM-CRFs model customized using fine-tuning strategy achieved the best strict and relaxed F1 scores of 0.9288 and 0.9584, respectively. The customized model significantly outperformed the LSTM-CRFs model trained only using the i2b2 dataset (strict and relaxed F1 scores of 0.8736 and 0.9197, respectively) and another LSTM-CRFs model trained only using UF data (strict and relax F1 scores of 0.9195 and 0.9468, respectively). This study demonstrated that it is necessary to customize deep learning-based de-identification models when applied in cross-institute settings.

This study is different from previous studies where the training and test data were extracted from the same source with only a few note types [1, 6]. In this study, we used the 2014 i2b2/UTHealth de-identification corpus as the training dataset for model development and evaluated the performance using another corpus developed at UF health. Here, our goal is to examine a state-of-the-art deep learning-based de-identification method at a cross-institute setting, identify the bottlenecks and provide potential solutions. The baseline LSTM-CRFs model achieved good strict and relaxed F1-scores of 0.9547 and 0.9646 when the training and test data are from the same source. Whereas, the performance dropped remarkably when it was directly applied to the UF test dataset (0.8568 and 0.8958, respectively). After adding extra lexical features and knowledge features, the performance improved. We then sought to further customize the models using local resources (i.e., clinical notes from UF Health) and compared two different strategies for customization. The experimental results show that the LSTM-CRFs model customized using the UF data through fine-tuning achieved the best performance, which is a potential solution for de-identification systems in cross-institute settings.

We compared five different embeddings trained from the general English text, clinical text, and biomedical literature. The experimental results show that the CommonCrawl, a general English corpus-based word embeddings, achieved a better performance for de-identification compared to other embeddings trained from de-identified clinical text from MIMIC III database or biomedical literature. This finding is different from our previous studies of applying deep learning models for medical concepts, where the embeddings trained from clinical text is often the best choice. This is not surprising as the PHIs from MIMIC III notes have been removed by a de-identification procedure. Therefore, many PHIs from the input text were not found from the MIMIC embeddings. The CommonCrawl embeddings, on the other hand, were able to capture some PHIs such as names, dates, IDs, and addresses.

We compared two strategies, including merging corpora and fine-tuning, to customize the de-identification models using UF Health clinical notes. Both customization strategies outperformed the models trained using only the i2b2 data or only the UF data. The merging corpora strategy achieved comparable performance as the fine-tuning strategy in terms of micro-averaged F1 scores. However, the fine-tuning could re-use the pre-trained parameters and weights from a developed model and reduce the training time, which could be a better solution for customization of de-identification models in cross-institute settings.

## Conclusion

In this study, we explored a state-of-the-art deep learning method for de-identification of clinical notes at cross-institute settings. We compared five different word embeddings and two customization strategies, identified the bottlenecks, and provided potential solutions. This study demonstrated that deep learning-based de-identification methods could achieve a decent performance at cross-institute settings through customization using local resources.

## Data Availability

The 2014 i2b2/UTHealth Shared-Tasks and Workshop on Challenges in Natural Language Processing for Clinical Data Track 1 – de-identification dataset is available at https://www.i2b2.org/NLP/DataSets/.

## Abbreviations

CDW: Clinical Data Warehouse
CEGS: Centers of Excellence in Genomic Science
CNN: Convolutional Neural Networks
CRFs: Conditional Random Fields
EHR: Electronic Health Records
EMR: Electronic Medical Record
HIPAA: Health Insurance Portability and Accountability Act
I2B2: Informatics for Integrating Biology and the Bedside
IDR: Integrated Data Repository
LSTM: Long-Short Term Memory
ME: Maximum Entropy
MIMIC-III: Medical Information Mart for Intensive Care III
NER: Named Entity Recognition
N-GRID: Neuropsychiatric Genome-Scale and RDOC Individualized Domains
NLP: Natural Language Processing
PHI: Protected Health Information
RNN: Recurrent Neural Networks
SSVMs: Structured Support Vector Machines
UF: University of Florida

## References

[1] Meystre SM, Friedlin FJ, South BR, Shen S, Samore MH (2010). Automatic de-identification of textual documents in the electronic health record: a review of recent research. BMC Med Res Methodol.

[2] Kayaalp M (2018). Patient privacy in the era of big data. Balkan Med J.

[3] Kayaalp M, Browne AC, Sagan P, McGee T, McDonald CJ (2015). Challenges and insights in using HIPAA privacy rule for clinical text annotation. AMIA Annu Symp Proc..

[4] South BR, Mowery D, Suo Y, Leng J, Ferrández Ó, Meystre SM (2014). Evaluating the effects of machine pre-annotation and an interactive annotation interface on manual de-identification of clinical text. J Biomed Inform.

[5] Dorr DA, Phillips WF, Phansalkar S, Sims SA, Hurdle JF (2018). Assessing the difficulty and time cost of De-identification in clinical narratives. Methods Inf Med.

[6] Yogarajan V, Mayo M, Pfahringer B. A survey of automatic de-identification of longitudinal clinical narratives. CoRR. 2018;abs/1810.06765.

[7] Uzuner O, Luo Y, Szolovits P (2007). Evaluating the state-of-the-art in automatic de-identification. J Am Med Inform Assoc.

[8] Neamatullah I, Douglass MM, Lehman LH, Reisner A, Villarroel M, Long WJ (2008). Automated de-identification of free-text medical records. BMC Med Inform Decision Making.

[9] Stubbs A, Uzuner Ö (2015). Annotating longitudinal clinical narratives for de-identification: the 2014 i2b2/UTHealth corpus. J Biomed Inform.

[10] Stubbs A, Filannino M, Uzuner Ö (2017). De-identification of psychiatric intake records: overview of 2016 CEGS N-GRID shared tasks track 1. J Biomed Inform.

[11] Ferrández Ó, South BR, Shen S, Friedlin FJ, Samore MH, Meystre SM (2012). Generalizability and comparison of automatic clinical text de-identification methods and resources. AMIA Annu Symp Proc.

[12] Ferrández O, South BR, Shen S, Friedlin FJ, Samore MH, Meystre SM (2012). Evaluating current automatic de-identification methods with Veteran’s health administration clinical documents. BMC Med Res Methodol.

[13] Zuccon G, Kotzur D, Nguyen A, Bergheim A (2014). De-identification of health records using anonym: effectiveness and robustness across datasets. Artif Intell Med.

[14] Nadkarni PM, Ohno-Machado L, Chapman WW (2011). Natural language processing: an introduction. J Am Med Inform Assoc.

[15] Stubbs A, Kotfila C, Uzuner Ö (2015). Automated systems for the de-identification of longitudinal clinical narratives: overview of 2014 i2b2/UTHealth shared task track 1. J Biomed Inform.

[16] Lafferty JD, McCallum A, Pereira FCN (2001). Conditional random fields: probabilistic models for segmenting and labeling sequence data. Proceedings of the eighteenth international conference on machine learning.

[17] Tsochantaridis I, Joachims T, Hofmann T, Altun Y (2005). Large margin methods for structured and interdependent output variables. J Mach Learn Res.

[18] Yang H, Garibaldi JM (2015). Automatic detection of protected health information from clinic narratives. J Biomed Inform.

[19] Liu Z, Chen Y, Tang B, Wang X, Chen Q, Li H (2015). Automatic de-identification of electronic medical records using token-level and character-level conditional random fields. J Biomed Inform.

[20] Lee H-J, Wu Y, Zhang Y, Xu J, Xu H, Roberts K (2017). A hybrid approach to automatic de-identification of psychiatric notes. J Biomed Inform.

[21] Shickel B, Tighe PJ, Bihorac A, Rashidi P (2018). Deep EHR: a survey of recent advances in deep learning techniques for electronic health record (EHR) analysis. IEEE J Biomed Health Inform.

[22] Xiao C, Choi E, Sun J (2018). Opportunities and challenges in developing deep learning models using electronic health records data: a systematic review. J Am Med Inform Assoc.

[23] Wu Y, Jiang M, Lei J, Xu H (2015). Named entity recognition in Chinese clinical text using deep neural network. Stud Health Technol Inform.

[24] Wu Y, Jiang M, Xu J, Zhi D, Xu H (2017). Clinical named entity recognition using deep learning models. AMIA Annu Symp Proc..

[25] Wu Y, Yang X, Bian J, Guo Y, Xu H, Hogan W (2018). Combine factual medical knowledge and distributed word representation to improve clinical named entity recognition. AMIA Ann Symp Proc.

[26] Yang X, Bian J, Gong Y, Hogan WR, Wu Y. MADEx: a system for detecting medications, adverse drug events, and their relations from clinical notes. Drug Saf. 2019. 10.1007/s40264-018-0761-0. 10.1007/s40264-018-0761-0 PMC6402874 30600484

[27] Liu Z, Tang B, Wang X, Chen Q (2017). De-identification of clinical notes via recurrent neural network and conditional random field. J Biomed Inform.

[28] Lee JY, Szolovits P, Dernoncourt F, Uzuner O (2016). De-identification of patient notes with recurrent neural networks. J Am Med Inform Assoc.

[29] Ramshaw L, Marcus M. Text chunking using transformation-based learning. In: Third Workshop on Very Large Corpora. 1995. http://aclweb.org/anthology/W95-0107.

[30] Jiang Z, Zhao C, He B, Guan Y, Jiang J (2017). De-identification of medical records using conditional random fields and long short-term memory networks. J Biomed Inform.

[31] Jiang M, Chen Y, Liu M, Rosenbloom ST, Mani S, Denny JC (2011). A study of machine-learning-based approaches to extract clinical entities and their assertions from discharge summaries. J Am Med Inform Assoc.

[32] Reimers N, Gurevych I. Optimal Hyperparameters for Deep LSTM-Networks for Sequence Labeling Tasks. CoRR. 2017;abs/1707.06799. http://arxiv.org/abs/1707.06799.

[33] Mikolov T, Sutskever I, Chen K, Corrado G, Dean J (2013). Distributed representations of words and phrases and their compositionality. Proceedings of the 26th international conference on neural information processing systems - volume 2.

[34] Joulin A, Grave E, Bojanowski P, Douze M, Jégou H, Mikolov T. FastText.zip: Compressing text classification models. arXiv preprint arXiv. 2016:161203651.

[35] Mikolov T, Grave E, Bojanowski P, Puhrsch C, Joulin A. Advances in Pre-Training Distributed Word Representations. CoRR. 2017;abs/1712.09405.

[36] Johnson AEW, Pollard TJ, Shen L, Lehman LH, Feng M, Ghassemi M (2016). MIMIC-III, a freely accessible critical care database. Scientific Data.

[37] Jagannatha A, Yu H. Structured prediction models for RNN based sequence labeling in clinical text. CoRR. 2016;abs/1608.00612. http://arxiv.org/abs/1608.00612. 10.18653/v1/d16-1082 PMC5167535 28004040

[38] Howard J, Ruder S. Universal language model fine-tuning for text classification. In: ACL. 2018.

[39] Dai AM, Le QV. Semi-supervised sequence learning. In: NIPS. 2015.

[40] Radford A, Narasimhan K, Salimans T, Sutskever I. Improving language understanding by generative pre-training.

[41] Martín Abadi, Ashish Agarwal, Paul Barham, Eugene Brevdo, Zhifeng Chen, Craig Citro, et al. TensorFlow: Large-Scale Machine Learning on Heterogeneous Systems. 2015. https://www.tensorflow.org/.

